# Validation of the Portuguese Version of the Fertility Adjustment Scale

**DOI:** 10.3390/healthcare10030563

**Published:** 2022-03-17

**Authors:** Joana Romeiro, Paulo Nogueira, Sílvia Caldeira

**Affiliations:** 1 Institute of Health Sciences, Centre for Interdisciplinary Research in Health, Palma de Cima, Catholic University of Portugal, 1649-023 Lisbon, Portugal; scaldeira@ucp.pt; 2 Faculdade de Medicina, Instituto de Medicina Preventiva e Saúde Pública, Universidade de Lisboa, 1649-028 Lisboa, Portugal; paulo.nogueira@edu.ulisboa.pt

**Keywords:** adjustment, assisted reproductive techniques, infertility, validation studies

## Abstract

There is an urgent need to provide healthcare professionals and midwives with validated tools as to improve fertility adjustment and promote well-being of couples with infertility. The purpose of this study was to test validity of the Fertility Adjustment Scale among people undergoing assisted reproductive techniques. A cross-sectional and methodological study was conducted, and a total of 104 Portuguese adults undergoing fertility treatment were recruited through fertility-related websites. The Fertility Adjustment Scale was administered along with the Spiritual Well-Being Questionnaire and the Resilience Scale for adults as a measure of concurrent validity. Scores revealed the sample’s lack of adjustment to fertility. A significant correlation with measures of resilience provided evidence of convergent validity. There was a significant association of fertility adjustment with time of consultation and the cause of infertility. A Fertility Adjustment Scale with six items is a reliable tool that offers early recognition of patients’ difficulties in adaptation to fertility problems during assisted reproductive techniques, which could be beneficial in not only an early recognition of healthcare intervention but of a more individualized approach to such patients.

## 1. Introduction

Infertility is a reproductive health condition related to a failure to have a clinical pregnancy in one year of regular, unprotected sexual intercourse and also described as an impaired ability of a person or a couple to reproduce [1]. Yet, this time frame is reduced to 6 months in persons over 35 years [1]. Two types of infertility have been clinically identified. Primary infertility occurs when there is inability of the couples to achieve conception and have a successful live birth. Secondary infertility happens when there is an inability to achieve conception and have a successful live birth in couples that previously had a successful pregnancy and a biological child [1].

Despite this scientific definition, infertility goes beyond a physical and objective condition. Indeed, early studies described that when involuntary childlessness was experienced, it was perceived as an adverse event experienced by individuals and couples [2]. Moreover, patients have described a profound and intense impact concomitant with a sense of having suffered multiple losses, starting with the loss of the natural and expected ability of conception, pregnancy, and birth, along with the frustrated wishes of motherhood and/or parenthood. In addition, strained relationships (between partners, extended family, friends, co-workers, others, self, and a higher power); financial difficulties (due to high treatment costs); and changes in daily routines, such as attending scheduled fertility consultations, exams, and treatment procedures, are common challenges endured by patients with infertility [2,3].

Consequently, infertility has been associated with deep changes in all life domains, including existential and quality of living [4]. As such, this phenomenon forces an individual to face a series of challenges and to adapt. At the heart of such adjustment is the unfulfilled wish of having offspring, which launches couples into a relentless and desperate pursuit for medical assistance and assisted reproductive techniques (ART). On this path, couples often described enduring long, painful, and consecutive treatments along with a mixture of feelings due to unsuccessful cycles of ART [5]. Ultimately, all personal, emotional, psychological, spiritual, social, and cultural dimensions have been described to be disturbed and thus raise the need to activate resources and skills to cope with such a reproductive phenomenon [6]. At this point, psychological adjustment takes a key role. Psychological adjustment is related to the cognitive, behavioral, and emotional aspects in which individuals process, recognize, and deal with life events [7]. Several degrees of adjustment to fertility might be lived during different stages of treatment, and a higher stage of adjustment does not necessarily mean passivity or resignation to childlessness. Instead, it relates to an individual being able to process any possible outcome (being able or not being able to have a child) [7].

ART can be a means to achieve parenting, but the rate of success is limited and may lead to frustrating couples’ expectations, with high rates of failure around 75%, such as is the case of in vitro fertilization (IVF) [8]. Therefore, an effective adjustment to involuntary childlessness and infertility should be grounded on coping skills to overcome the hardness of consecutive failed treatments [9,10]. Indeed, a previous review of empirical evidence reported that relational and personal issues and psychological burdens were responsible for individuals’ treatment discontinuation [11]. Furthermore, one in every ten women living with infertility seems to have a compromised trajectory of adapting to stressors, which would cause serious mental health impairment years after treatment [9]. 

In this regard, reliable and valid instruments are essential for assessing and understanding patients’ needs, identifying psychological vulnerability, and screening patients’ adaptation to adversity. This is crucial in such a health context to prevent emotional distress, treatment desertion, and mental health problems. Therefore, mapping adaptation to fertility is key to promote patient’s coping strategies to face failed cycles as well as enhance treatment compliance (and increase chances of success) and promote couples’ positive mental health and psychological well-being [12,13,14].

In this regard, a specific tool named Fertility Adjustment Scale (FAS) was developed by Glover and collaborators [7], and its original structure of 12 items has been the target of cultural adaptation and validation in some countries [15,16]. The Portuguese version was introduced by Lopes and Leal [17,18] and tested in 35 women and 35 men attending infertility consultations. No other known research study has attempted to test the psychometric proprieties, validity, and reliability of this 10-item Portuguese version. Therefore, the purpose of this study was to test validity of the Fertility Adjustment Scale among people undergoing assisted reproductive techniques. Furthermore, associations were analyzed between FAS and sociodemographic and clinical variables. 

## 2. Materials and Methods

This cross-sectional and methodological study was undertaken using a self-administered online questionnaire. Inclusion criteria were adult women or men (18 years or older) with infertility. Because reports of this experience might not vividly or accurately be described when women or men were not under ART, only targeted individuals going through fertility treatment or at the eminence of ART during data collection were included. Incomplete questionnaires and respondents that were not Portuguese citizens were excluded.

Recruitment was based on fertility-related websites and gathered a non-probabilistic sample of 104 Portuguese adults (18 years or older) in the process of engaging in or at any stage of a fertility treatment. 

Before its implementation, the questionnaire was submitted for consideration by three nursing experts, followed by a pretest with a sample of thirty respondents recruited by the same means. Afterwards, preliminary findings and suggestions made by the experts led to minor changes to provide more comprehensive and clearer writing. Participants were fully informed about the aims and the study process, and all gave their informed consent before being granted access to the online questionnaire.

The current study is part of the first author’s doctoral thesis and was approved by The Ethics Committee of The Institute of Health Sciences of Universidade Católica Portuguesa (certificate dates 13 March 2019).

The survey completed by the participants comprised sociodemographic information as well as spiritual/religious details and clinical health data. The Portuguese version of the FAS comprises a 10-item structure [17,18] and accommodates three subscales: “*centrality of parenting*” (including items 1, 3, 6, and 9); “*suspended life*” (items 2, 5, and 8); and “*acceptance of life without children*” (items 4, 7, and 10). A six-point Likert scale is used to score each item (ranging from 1 = “*strongly disagree*” to 6 = “*strongly agree*”). The total score of the overall instrument ranges from 10 to 60, and higher scores indicate lower levels of fertility adjustment. The total Cronbach’s alpha (α) of the original Portuguese version was 0.80 [17,18].

Statistical analyses were conducted using SPSS, version 26.0 [19]. 

Demographic and clinical data were presented as means and standard deviations (SD) or absolute frequencies and percentages. The Independent sample *t*-tests and the One-Way ANOVA were used to detect differences in FAS means between subgroups (Table 1) when data normality assumptions were met according to Pestana and Gageiro [20]. 

The internal consistency and reliability of the instrument were determined by calculating Cronbach’s alpha (higher than 0.70) [21]. The suitability to perform a factor analysis was obtained through Bartlett’s Test of Sphericity (*p* < 0.05) [22] and the Kaiser–Meyer–Olkin (KMO) sampling adequacy test (higher than 0.6) [23].

A confirmatory factor analysis (CFA) was first conducted using AMOS SPSS program (Analysis of Moment Structures), version 26.0 [19]. A good fit was achieved if goodness-of-fit indices presented the following recommended values: ratio of chi-square statistic to the respective degrees of freedom (*χ*^2^/df) (lower than 3); the root mean square error of approximation (RMSEA) with a value of 0.01 (excellent fit), below 0.05 (good fit) with lower than 0.08 also considered a good fit, between 0.05–0.10 (moderate fit), and higher than 0.10 (bad fit); the incremental fit index (CFI) (greater than 0.90–0.95); Tucker–Lewis Index (TLI) ranges between 0 and 1 with values greater than 0.90 indicating good fit; and Normed Fit Index (NFI) higher than 0.90 [24,25,26]. If bad-fit statistics were identified, further examination of the 10-item model [17,18] would be done through exploratory factor analysis (EFA) with a Varimax rotation. Items with communalities less than 0.2 were removed during this process. A principal component extraction performed based on eigenvalues greater than one (Kaiser criterion or K1) [27]. Factors with at least 3 items and a loading greater than 0.4 and a low cross-loading were retained. Additional scree plot analysis was used to analyze findings. 

Convergent and divergent validity of the final FAS structure was further investigated using Pearson’s correlation coefficient between each variable of the FAS and the Portuguese version of the Resilience Scale (RS) for adults [28] and the Spiritual Well-Being Questionnaire (SWBQp) [29]. 

## 3. Results

### 3.1. Sample Characteristics

A total of one hundred and four participants (*n* = 104) met the inclusion criteria for this study, and no respondent was excluded, as there were no missing or incomplete answers. The sample included 102 women (98.1%) and 2 men (1.9%). Age ranged from 26 to 54 years with a mean of 35.4 (SD = 4.8; 95% CI = 34.49–36.34). The majority of the sample lived in the northern region of Portugal (33.3%), were married (57.7%), and living together for a mean of 7.97 years (SD = 4.80; 95% CI = 7.03–8.91). Most had a higher education level (65.4%), were employed (87.5%), and were intellectual and scientific experts (35.6%). Much of the sample revealed a primary form of infertility (86.5%). Individuals were also dealing with infertility for a mean time of 3.63 years (SD = 3.33) and were followed in consultation for a mean of 3.10 years (SD = 3.31). It must be also highlighted the predominance of respondents that had previously submitted to treatments (*n* = 57; 54.8%) and were waiting to start another treatment cycle (*n* = 49; 47.1%).

### 3.2. Descriptive Statistics of FAS

Table 1 provides an overview of the mean scores obtained by specific groups within the study sample. 

Findings revealed a significant association of fertility adjustment with time of consultation (*p* = 0.031) and the cause of infertility (*p* = 0.038) (Table 1). Adjustment was decreased in people attending consultation between 7–9 years (M = 4.21; SD = 0.76) and in individuals still waiting for diagnosis results concerning infertility cause (M = 4.17; SD = 0.38). The items’ mean score for the FAS was 3.94 (SD = 0.70), and the total score for the overall scale was 39.48 (SD = 7.03) (Table 2).

The scale presented a Cronbach’s alpha of 0.51, indicating a lack of internal consistency or homogeneity of the FAS. As such, and since the 10-item tool presented a significant Bartlett’s test of Sphericity (*χ*^2^ = 366.253, df = 45, *p* = 0.000) and KMO value of 0.782, factor analysis was performed.

### 3.3. Confirmatory Factor Analysis

Despite the abovementioned results, structural validity of the Portuguese version of the FAS was first analyzed by means of a CFA. As it was expected, findings did not confirm the hypothesized structural model since a bad fit with the observed data was obtained (Table 3).

### 3.4. Exploratory Factor Analysis

The Portuguese version of the FAS was analyzed using an EFA and principal component analysis (PCA).

**Figure 1:**
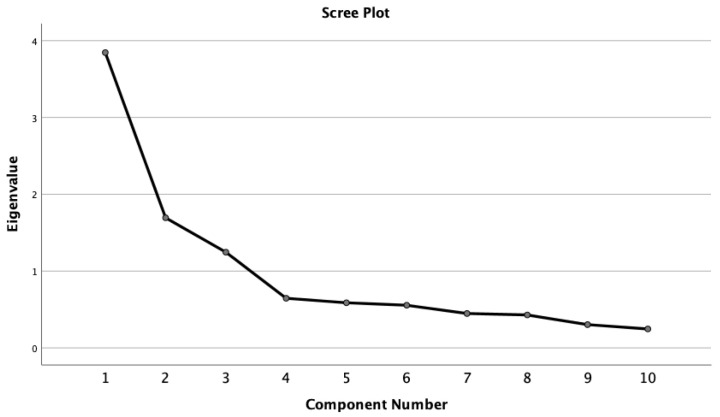
Scree plot representing the eigenvalues (10-item Fertility Adjustment Scale).

The K1 criterion (Table 4), along with the scree plot analysis (Figure 1), would seem to argue for a two- or three-factor solution although different from the original FAS model.

In addition, a Varimax rotation with K1 presented a scale structure with Factor 1 (items 1, 3, 6, and 9), Factor 2 (items 4, 7, 8, and 10), and Factor 3 (items 2 and 5) (Table 5). 

A factor with two variables is reliable only when the correlation between variables is high (*r* > 0.70) [30], and this was not the case since the correlation value between variables 2 and 5 in Factor 3 was 0.66. These findings raised questions about the three-factor solution.

Meanwhile, an item-total analysis reduced the number of variables included in the PCA to six items to improve the overall internal consistency (0.81). In this process, items 4, 7, 8, and 10 were excluded. 

At this point, the 6-item version of FAS was the focus of the analysis. The Bartlett’s test of Sphericity (*χ*^2^ = 226.245, df = 15, *p* < 0.001) and the KMO value of 0.754 revealed suitability to perform factor analysis. A PCA with Varimax rotation revealed a two-factor solution: Factor 1 (items 1, 3, 6, and 9) and Factor 2 (items 2 and 5) that explained 69.60% of the total variance (Table 2). The first component was labeled “*centrality of parenting*” and the second known as “*suspended life*”.

Although Factor 2 included only two variables (item 2 and 5), correlation between them was high and above the recommended value (0.72). Therefore, these items and the factor were not excluded from the FAS structure (Table 5).

Communalities initial values were equal to one and after extraction ranged between 0.63 and 0.74. No items were deleted, as recommended values after the extraction were above 0.3 (Table 6).

**Figure 2:**
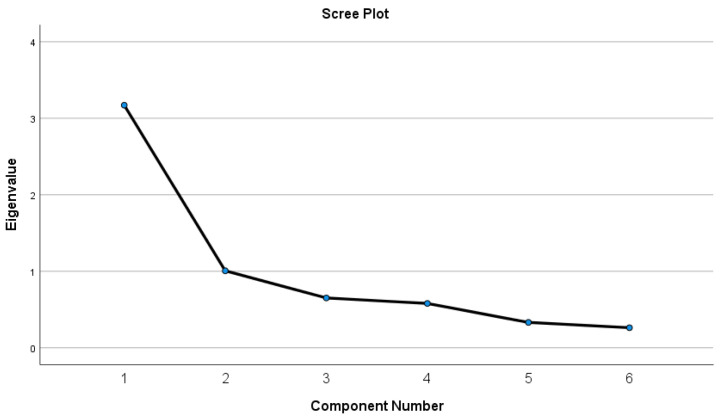
Scree plot representing the eigenvalues (6-item FAS).

Factor loadings of the 6-item tool ranged from 0.66 to 0.83, with recommended values above the cut-point of 0.30 (Table 3). No cross-loadings were observed. The scree plot (Figure 2) confirmed the two-factor structure of the 6-item FAS. 

Pearson’s correlation between Factor 1 and Factor 2 was significant (*r* = 0.48; *p* < 0.001) and above the recommended value (*r* ≥ 0.30). The 6-item scale presented a mean for the overall tool of 24.57 (SD = 7.27) and an item’s mean of 4.09 (SD = 1.21). Moreover, Factor 1 had a mean of 17.29 (SD = 5.41), and Factor 2 had a mean of 7.29 (SD = 2.91) (Table 7).

Skewness and kurtosis values for the two factors did not exceed the critical values of 1 and 2, respectively; as such, the normality assumption was met for the sample.

### 3.5. Reliability and Validity

The reconfigured FAS with six items presented a high internal consistency, with a Cronbach’s alpha of 0.82 (Factor 1 α = 0.82; Factor 2 α = 0.66).

The validity of the 6-item FAS was further investigated by comparing it to the SWBQp and the RS. Resilience and fertility adjustment have been theoretically related, and the hypothesis of a strong negative correlation between the two constructs was not statistically rejected in this study (Pearson coefficient (*r*) = −0.239; *p* = 0.015) (Table 5), which may be seen as an indication that convergent validity exists.

On the other hand, correlation of the reconfigured FAS with the SWBQp indicated discriminant validity (*r* = −0.128; *p* = 0.197), with most Pearson’s values indicating the uniqueness of the FAS although the “*suspended life*” factor had a significant and negative correlation with half of the SWBQp subscales (Table 8).

## 4. Discussion

The purpose of this study to test validity of the Fertility Adjustment Scale among people undergoing assisted reproductive techniques was accomplished. The reliability and validity of the Portuguese version of FAS [17,18] in a Portuguese sample of adults undergoing fertility treatment were herein determined. The use of the same measurement tool in a similar sample and context is not new. However, there is scarce evidence regarding tests of the psychometric properties of the FAS in the Portuguese population [17,18]. 

A CFA followed by an EFA was conducted and revealed that the three factors and 10-item scale were not supported by data. Further analysis of this structure suggested a 6-item instrument with two distinct dimensions: one designated as “*centrality of parenting*” and the other as “*suspended life*”. Therefore, the results of this study deviated from the 10-item and 3-factor structure as presented by Lopes and Leal [17,18]. This two-factor solution explained 69.60% of the total variance, higher than the 63.55% of the scale of Lopes and Leal [17].

Moreover, the FAS with six items revealed a high internal consistency, with a Cronbach’s alpha of 0.82 (Factor 1 = 0.82; Factor 2 = 0.66), and these values were also higher than the ones obtained by Lopes and Leal [17,18] (0.80; Factor 1 = 0.80; Factor 2 = 0.60). Still, the Cronbach’s alpha obtained in this study was lower than the 0.86 presented by Glover and collaborators [7]. 

Strong intercorrelations between factors of the FAS (*r* = 0.48; *p* < 0.001) were identified. Additionally, the FAS was significantly and negatively correlated to the RS (*r* = −0.239; *p* = 0.015). These are not surprising results. It confirms the known association between resilience and adjustment to fertility and the fact that resilient individuals demonstrated the effective adaptative process [31] to fertility conditioning and childlessness. The lack of correlation of the FAS with the SWBQp (*r* = −0.128; *p* = 0.197) suggests that the two measurement tools evaluate different and structurally independent constructs. Furthermore, slightly high values of the item’s mean (M = 4.09; SD = 1.21) and the mean for the overall tool (M = 24.57; SD = 7.27) demonstrated a lack of adjustment in this sample. These findings and the predominance of individuals who submitted to previous treatment (*n* = 57; 54.8%) reinforce what was stated by Moura-Ramos and collaborators [32] about previous cycles inducing negative consequences in emotional adjustment of individuals during fertility treatment. These also re in line with findings of a systematic review regarding women’s adjustment to ART [33], which identified acceptance of childlessness as a relevant predictor of emotional response to treatment. Since most of our respondents were women, these results have reinforced preliminary findings from previous research that revealed that women believed in treatment success and accepted ART more easily than men [31]. Instead, men would accept more often being childless when compared to women [31].

On the other hand, this study highlighted the significant association between individuals’ adjustment to fertility and time of consultation (*p* = 0.032), with higher results pointing to a decreased adjustment in people followed between 7 to 9 years in consultation (M = 4.21; SD = 0.76). This fact might be explained by the inclusion of respondents searching for ART or in the middle of a fertility treatment. This persistent pursuit for treatment options in order to achieve the so-desired child has been linked with the likelihood for an absent process of dealing with loss and a lack of adaptation to definitive childlessness [33]. Such arguments might reflect the elimination of one of the original factors (“*acceptance of life without children*”) of the FAS presented by Glover and collaborators [7] and also adopted by Lopes and Leal [17,18]. 

Finally, a significant association was also found between adjustment and the cause of infertility (*p* = 0.038), with a higher score obtained in individuals who were still unaware of the cause for not having offspring (M = 4.17; SD = 0.38). If individuals do not have an objective cause for understanding infertility, then the cognitive process that Verhaaket and collaborators [33] argued necessary for adjustment to unsuccessful treatment might be compromised. 

The following limitations should be considered when reading the results: the major involvement of women and few men in the study could bias the overall results of the sample; recruitment happened through the web, and as such, the sample included only participants with internet access, which could have compromised findings; implementation of the online questionnaire occurred in a more extended period of time than was expected or scheduled due to a slow adherence of respondents. This might be explained by the simultaneous recruitment method used by other scholars at the same time as the sudden onset of the COVID-19 pandemic. Indeed, Sathian and collaborators [34] argued through a literature review that COVID-19 had a great impact on research and was found to be accountable for timeline delays and, as such, for possible outcomes bias. In fact, the devastating impact of COVID-19 on the worldwide population [35] led governments to impose restrictions to manage the healthcare system [34]. Due to that, one might also extrapolate possible bias in this study’s results added to Portuguese individuals dealing with infertility as reproductive assistance since ART were postponed [34].

## 5. Conclusions

In conclusion, our findings showed that the shortened, Portuguese version of the FAS with six items can be used in individuals going through ART. 

In this time of enhanced concerns about couples’ fertility issues and treatments, the FAS could be useful in identifying patients’ psychological needs and therefore allowing an early, patient-centered intervention directed towards adjustment to fertility problems at different stages of ART. These will aid patients in the process of becoming resilient, regaining control over their lives, developing realistic expectations, dealing with both positive and negative treatment outcomes, and preventing adverse mental health outcomes and treatment withdrawals. 

Further studies concerning the adaptative processes in both men and women or couples are needed. The use of the same methodology by other researchers could provide enough evidence to allow comparisons. Finally, testing the reliability and validity of the FAS along with psychometric properties of the shortened version as presented in this paper in similar samples and distinct populations will help to build a body of knowledge capable of warranting the robustness of this tool in measuring adjustment to fertility.

## Figures and Tables

**Table 1:** Mean scores of the FAS regarding characteristics of participants (*n* = 104).

Variable		FAS		
		Response Mean (SD)	*p*	Total Mean (SD)
Social—Demographic				
Gender			0.080 ^a^	
	Female	3.94 (0.71)		39.49 (7.10)
	Male	3.90 (0.00)		39.00 (0.00)
Age			0.902 ^b^	
	≤34	4.05 (0.71)		40.53 (7.13)
	35–40	4.01 (0.62)		40.17 (6.26)
	41–51	3.30 (0.66)		33.09 (6.65)
	≥52	3.00 (0.00)		30.00 (0.00)
Marital Status			0.768 ^b^	
	Married	4.00 (0.71)		40.06 (7.11)
	Together	3.89 (0.56)		38.97 (7.21)
	Divorced/Separated	3.73 (0.45)		36.33 (5.68)
	Single	3.73 (0.45)		37.33 (4.50)
Current relationship (years)			0.890 ^b^	
	≤3	3.98 (0.62)		39.88 (6.26)
	4–6	4.04 (0.65)		40.44 (6.50)
	7–9	4.05 (0.75)		40.53 (7.57)
	≥10	3.75 (0.73)		37.58 (7.37)
Education level			0.956 ^b^	
	Middle school	3.90 (0.00)		39.00 (0.00)
	High school	3.98 (0.67)		39.80 (6.74)
	Professional course	4.15 (0.79)		41.55 (7.90)
	Bachelor/Graduation	3.92 (0.72)		39.25 (7.23)
	Master’s	3.95 (0.65)		39.52 (6.50)
	Ph.D.	3.40 (0.96)		34.00 (9.64)
Employment status			0.353 ^b^	
	Employed	3.96 (0.70)		39.61 (7.03)
	Unemployed	3.96 (0.60)		39.63 (6.02)
	Student	3.25 (1.34)		32.50 (13.43)
Occupation			0.574 ^b^	
	Representatives of the legislative branch of executive bodies, officers, directors, and executive managers	3.74 (0.83)		37.40 (8.38)
	Experts from intellectual and scientific activities	4.09 (0.67)		40.94 (6.79)
	Intermediate-level technicians and professions	4.07 (0.33)		40.75 (3.30)
	Administrative staff	3.86 (0.61)		38.64 (6.18)
	Personal service, security, and safety workers and salespeople	3.92 (0.80)		39.22 (8.02)
	Skilled workers in industry, construction, and craftsmen	2.90 (0.00)		29.00 (0.00)
	Plant and machine operators	4.90 (0.00)		49.00 (0.00)
Spirituality—Religion				
Spiritual person			0.490 ^a^	
	No	4.01 (0.64)		40.16 (6.40)
	Yes	3.92 (0.72)		39.26 (7.24)
Spiritual importance			0.745 ^b^	
	Not important	4.03 (0.59)		40.36 (5.98)
	Little importance	3.83 (0.72)		38.39 (7.22)
	Important	3.94 (0.70)		39.47 (7.05)
	Very important	4.17 (0.84)		41.71 (8.45)
Spiritual change with diagnosis			0.501 ^b^	
	No change	4.03 (0.70)		40.34 (7.07)
	Less important	3.72 (0.82)		37.29 (8.29)
	More important	3.92 (0.62)		39.25 (6.21)
Spiritual change with treatment			0.360 ^b^	
	No change	3.94 (0.75)		39.49 (7.57)
	Less important	3.80 (0.80)		38.00 (8.00)
	More important	4.00 (0.57)		40.08 (5.77)
Religious person			0.181 ^a^	
	No	3.78 (0.59)		37.82 (5.92)
	Yes	4.02 (0.74)		40.28 (7.41)
Religion importance			0.745 ^b^	
	Not important	4.03 (0.59)		40.36 (5.98)
	Little importance	3.83 (0.72)		38.39 (7.22)
	Important	3.94 (0.70)		39.47 (7.05)
	Very important	4.17 (0.84)		41.71 (8.45)
Religion changes with diagnosis			0.372 ^b^	
	No change	4.04 (0.71)		40.42 (7.12)
	Less important	3.82 (0.75)		38.28 (7.56)
	More important	3.90 (0.62)		39.00 (6.27)
Religion changes with treatment			0.066 ^b^	
	No change	3.98 (0.79)		39.85 (7.91)
	Less important	3.90 (0.75)		39.08 (7.59)
	More important	3.92 (0.52)		39.21 (5.20)
Clinical—Infertility				
Type			0.979 ^a^	
	Primary	3.99 (0.68)		39.95 (6.84)
	Secondary	3.64 (0.77)		36.42 (7.71)
Nature			0.179 ^b^	
	Never been pregnant	4.02 (7.59)		40.22 (7.59)
	Natural pregnancy without live birth	3.76 (0.57)		37.66 (5.78))
	Natural pregnancy, had child, not able to have another child	3.83 (0.70)		38.33 (7.01)
	Pregnancy with treatment, did not have a child	4.17 (0.36)		41.78 (3.62)
	Pregnancy with treatment, had child, not able to have another child	3.30 (0.84)		33.00 (8.48)
Cause			0.038 ^b^	
	Female	3.75 (0.83)		37.52 (8.30)
	Male	4.11 (0.42)		41.13 (4.20)
	Mixed	4.13 (0.71)		41.38 (7.11)
	Unknown	3.98 (0.58)		39.83 (5.82)
	Waiting diagnosis	4.17 (0.38)		41.77 (3.86)
Diagnosis (years)			0.052 ^b^	
	≤3	3.94 (0.62)		39.40 (6.22)
	4–6	3.98 (0.78)		39.80 (8.93)
	7–9	3.97 (0.89)		39.70 (8.93)
	≥10	3.97 (1.01)		39.71 (10.14)
Consultation (years)			0.031 ^b^	
	≤3	3.95 (0.62)		39.55 (6.24)
	4–6	3.85 (0.87)		38.57 (8.73)
	7–9	4.21 (0.76)		42.10 (7.68)
	≥10	3.66 (1.20)		36.60 (12.03)
**Treatments**				
Previous treatments			0.948 ^a^	
	No	4.01 (0.71)		40.12 (7.11)
	Yes	3.89 (0.69)		38.94 (6.98)
Time in current treatment (months)				
	≤3	3.66 (0.23)		36.66 (2.30)
	4–6	3.87 (0.51)		38.75 (5.12)
	7–12	3.79 (0.71)		37.93 (7.19)
	13–24	4.19 (0.73)		41.92 (7.33)
	24–36	3.76 (0.55)		37.60 (5.50)
	≥37	3.84 (0.78)		38.43 (7.89)
Current treatment			0.228 ^b^	
	Previous tests	3.93 (0.82)		39.30 (8.26)
	Waiting to start	4.04 (0.61)		40.48 (6.13)
	In cycle	3.97 (0.61)		39.78 (6.18)
	OI	3.90 (0.00)	0.839 ^b^	39.00 (0.00)
	IUI	4.10 (0.42)		41.00 (4.24)
	IVF	3.81 (0.70)		38.10 (7.06)
	ICSI	4.22 (0.65)		42.25 (6.50)
	Other	4.25 (0.49)		42.50 (4.94)
	Tests after cycle	3.62 (0.84)		39.48 (7.03)

Legend: ^a^ Independent Sample Student’s *t*-test (Levene’s Test); ^b^ One Way ANOVA test; SD, standard deviation; OI, ovulation induction; IUI, intrauterine insemination; IVF, in vitro fertilization; ICSI, intracytoplasmic sperm injection.

**Table 2:** Descriptive statistics, Cronbach´s alpha coefficient of FAS, and EFA assessment of normality of items.

Item	Mean	95% CI	SD	Skewness	Std. Error	Kurtosis	Std. Error	Cronbach Alpha (without Item)
1.	4.50	4.19–4.82	1.61	−0.913	0.237	−0.299	0.469	(0.400)
2.	3.35	3.00–3.70	1.76	−0.015	0.237	−1.336	0.469	(0.440)
3.	4.61	4.27–4.94	1.66	−1.038	0.237	−0.180	0.469	(0.403)
4.	3.62	3.32–3.94	1.62	−0.266	0.237	−1.065	0.469	(0.517)
5.	3.94	3.62–4.26	1.60	−0.329	0.237	−0.962	0.469	(0.400)
6.	4.33	3.99–4.65	1.69	−0.685	0.237	−0.746	0.469	(0.429)
7.	3.42	3.10–3.75	1.61	−0.028	0.237	−1.135	0.469	(0.558)
8.	4.33	4.03–4.58	1.42	−0.701	0.237	−0.256	0.469	(0.521)
9.	3.86	3.54–4.22	1.73	−0.329	0.237	−1.198	0.469	(0.480)
10.	3.54	3.22–3.86	1.65	−0.030	0.237	−1.113	0.469	(0.569)
**Total**	39.48	38.20–40.91	7.03	−0.275	0.237	0.183	0.469	0.505

CI, confidence interval; SD, standard deviation.

**Table 3:** CFA goodness-of-fit indices for the FAS models (*n* = 104).

Models	*χ*	df	*χ*/df	*p*	RMSEA (90%CI)	CFI	TLI	NFI
10-item 3-factor [17,18]	84.608	32	2.644	0.000	0.126 (0.094–0.159)	0.844	0.780	0.778
6-item 2-factor	28.402	8	3.550	0.000	0.157 (0.097–0.222)	0.906	0.824	0.878
6-item 1-factor	40.594	9	4.510	0.000	0.185 (0.129–0.244)	0.855	0.758	0.826

CFI, Comparative Fit Index; CI, confidence intervals; df, degrees of freedom; NFI, Normed Fit Index; RMSEA, Root-Mean-Square Error of Approximation; TLI, Tucker–Lewis Index; *χ*^2^, chi-square statistic; *χ*^2^/df, ratio of the differences in chi-square to the differences in degrees of freedom.

**Table 4:** Components extraction from data based on K1 criterion and total variance explained for FAS factor structure.

Components Extraction Based on K1 Criterion and Percentage (%) of Variance						
Component	Three-Factor (10 Items)			Two-Factor (6 Items)		
	Total	% Variance	Cumulative %	Total	% Variance	Cumulative %
1	3.844	38.444	38.444	3.170	52.840	52.840
2	1.694	16.939	55.383	1.006	16.762	69.602
3	1.246	12.460	67.843			

**Table 5:** Value of Varimax rotation factor loading of the FAS ^a^.

Varimax Rotation Factor Loading					
	Three-Factor (10 Items)			Two-Factor (6 Items)	
Components	1	2	3	1	2
1	0.84			0.80	
2			0.81		0.83
3	0.78			0.77	
4		0.74			
5			0.75		0.82
6	0.57			0.66	
7		0.75			
8		0.64			
9	0.68			0.81	
10		0.74			

^a^ For clarity, loadings < 0.5 are not shown.

**Table 6:** Communalities.

Communalities			
Variables		10-Item	6-Item 2-Factor
	Initial	Extraction	Extraction
1.	1.000	0.73	0.68
2.	1.000	0.68	0.71
3.	1.000	0.74	0.73
4.	1.000	0.61	
5.	1.000	0.69	0.74
6.	1.000	0.64	0.63
7.	1.000	0.63	
8.	1.000	0.80	
9.	1.000	0.60	0.67
10.	1.000	0.63	

**Table 7:** Descriptive Statistics of 6-item FAS factors.

Factor		Skewness			Kurtosis	
	Total Mean	SD	Statistic	SD	Statistic	Std. Error
1	3.64	1.45	−0.196	0.237	−0.861	0.469
2	4.32	1.35	−0.812	0.237	−0.067	0.469

**Table 8:** Discriminant validity of the reconfigured Resilience Scale factors.

Discriminant Validity of the Reconfigured FAS Factors						
	FAS Total		FAS—Factor 1 Centrality of Parenting		FAS—Factor 2 Suspended Life	
	*r*	*p*	*r*	*p*	*r*	*p*
**SWBQp variables**	−0.128	0.197	−0.022	0.823	−0.278 **	0.004
Personal	−0.248 *	0.011	−0.121	0.220	−0.397 **	0.000
Communal	−0.027	0.785	0.059	0.554	−0.177	0.072
Environmental	−0.170	0.084	−0.123	0.214	0.198 *	0.044
Transcendental	0.002	0.982	0.095	0.339	−0.170	0.084
**RS variables**	−0.239 *	0.015	−0.057	0.568	−0.336 **	0.000
Perseverance	−0.139	0.159	−0.134	0.176	−0.243 *	0.013
Meaning of life	−0.229 *	0.020	−0.163	0.099	−0.324 **	0.001
Serenity	−0.265 **	0.006	−0.184	0.061	−0.362 **	0.000
Self-reliance and self-confidence	−0.252 **	0.010	−0.141	0.153	−0.290 **	0.003

Legend: ** Correlation is significant at the 0.01 level (2-tailed); * Correlation is significant at the 0.05 level (2-tailed); *r*, Pearson coefficient.

## Data Availability

Not applicable.

## References

[1] Zegers-Hochschild F., Adamson G.D., Dyer S., Racowsky C., de Mouzon J., Sokol R., Rienzi L., Sunde A., Schmidt L., Cooke I.D. (2017). The International Glossary on Infertility and Fertility Care, 2017. Fertil. Steril..

[2] Romeiro J., Caldeira S. (2018). The Human Responses and Nursing Diagnoses of Those Living With Infertility: A Qualitative Systematic Review. Int. J. Nurs. Knowl..

[3] Greil A.L., Slauson-Blevins K., McQuillan J. (2010). The Experience of Infertility: A Review of Recent Literature. Sociol. Health Illn..

[4] Agostini F., Monti F., Andrei F., Paterlini M., Palomba S., La Sala G.B. (2017). Assisted Reproductive Technology Treatments and Quality of Life: A Longitudinal Study among Subfertile Women and Men. J. Assist. Reprod. Genet..

[5] Cabral H., Bucher-Maluschke J. (2016). Development of Resilience Contribution in the Infertility Context: Review. Psicol. Saúde E. Doenças.

[6] Peters K., Jackson D., Rudge T. (2011). Surviving the Adversity of Childlessness: Fostering Resilience in Couples. Contemp. Nurse.

[7] Glover L., Hunter M., Richards J.M., Katz M., Abel P.D. (1999). Development of the Fertility Adjustment Scale. Fertil. Steril..

[8] Chochovski J., Moss S.A., Charman D.P. (2013). Recovery after Unsuccessful in Vitro Fertilization: The Complex Role of Resilience and Marital Relationships. J. Psychosom Obs. Gynaecol..

[9] Gameiro S., van den Belt-Dusebout A.W., Smeenk J.M.J., Braat D.D.M., van Leeuwen F.E., Verhaak C.M. (2016). Women’s Adjustment Trajectories during IVF and Impact on Mental Health 11–17 Years Later. Hum. Reprod..

[10] Iordăchescu D.A., Paica C.I., Boca A.E., Gică C., Panaitescu A.M., Peltecu G., Veduță A., Gică N. (2021). Anxiety, Difficulties, and Coping of Infertile Women. Healthcare.

[11] Gameiro S., Boivin J., Peronace L., Verhaak C.M. (2012). Why Do Patients Discontinue Fertility Treatment? A Systematic Review of Reasons and Predictors of Discontinuation in Fertility Treatment. Hum. Reprod. Update.

[12] Boivin J., Gameiro S. (2015). Evolution of Psychology and Counseling in Infertility. Fertil. Steril..

[13] Gameiro S., Verhaak C.M., Kremer J.A., Boivin J. (2013). Why We Should Talk about Compliance with Assisted Reproductive Technologies (ART): A Systematic Review and Meta-Analysis of ART Compliance Rates. Hum. Reprod. Update.

[14] Zurlo M.C., Cattaneo Della Volta M.F., Vallone F. (2018). Predictors of Quality of Life and Psychological Health in Infertile Couples: The Moderating Role of Duration of Infertility. Qual. Life Res..

[15] Tiyuri A., Vagharseyyedin S.A., Torshizi M., Bahramian N., Hajihosseini M. (2018). The Persian Version of Fertility Adjustment Scale: Psychometric Properties. Int. J. Fertil. Steril..

[16] Torabi M., Kazemi A., Abdishahshahani M. (2019). Psychometric Properties of Revised Version of the Fertility Adjustment Scale in Infertile Couples Undergoing Assisted Reproductive Technology. Eur. J. Obstet. Gynecol. Reprod. Biol..

[17] Lopes V., Leal I. (2010). Avaliação em Sexualidade e Parentalidade—Escala de Ajustamento à Fertilidade (EAF).

[18] Lopes V.M.S. (2008). Personalidade e Ajustamento Emocional na Infertilidade. Master’s Thesis.

[19] IBM SPSS Version 26.0. https://www.ibm.com/us-en/.

[20] Pestana M.H., Gageiro J.N. (2003). Análise de Dados Para Ciências Sociais: A Complementariedade do SPSS.

[21] Nunnally J.C., Bernstein I.H. (1994). The Assessment of Reliability. Psychometric Theory.

[22] Hair J.F., Black W.C., Babin B.J., Anderson R.E. (2010). Multivariate Data Analysis.

[23] Tabachnick B.G., Fidell L.S. (2007). Using Multivariate Statistics.

[24] Awang Z. (2012). The second order Confirmatory Factor Analysis. A Handbook on SEM.

[25] Hooper D., Coughlan J., Mullen M. (2008). Structural Equation Modelling: Guidelines for Determining Model Fit. Electron. J. Bus. Res. Methods.

[26] Hu L., Bentler P.M. (1998). Fit Indices in Covariance Structure Modeling: Sensitivity to Underparameterized Model Misspecification. Psychol. Methods.

[27] Hayton J.C., Allen D.G., Scarpello V. (2004). Factor Retention Decisions in Exploratory Factor Analysis: A Tutorial on Parallel Analysis. Organ. Res. Methods.

[28] de Carvalho Ng Deep C.A.F., Pereira I. (2012). Adaptação Da “The Resilience Scale” Para a População Adulta Portuguesa. Psicol. USP.

[29] Gomez R., Fisher J.W. (2003). Domains of Spiritual Well-Being and Development and Validation of the Spiritual Well-Being Questionnaire. Personal. Individ. Differ..

[30] Yong A.G., Pearce S. (2013). A Beginners Guide to Factor Analysis: Focusing on Exploratory Factor Analysis. Tutor. Quant. Methods Psychol..

[31] Nagórska M., Bartosiewicz A., Obrzut B., Darmochwał-Kolarz D. (2019). Gender Differences in the Experience of Infertility Concerning Polish Couples: Preliminary Research. Int. J. Environ. Res. Public Health.

[32] Moura-Ramos M., Gameiro S., Canavarro M.C., Soares I., Almeida-Santos T. (2016). Does Infertility History Affect the Emotional Adjustment of Couples Undergoing Assisted Reproduction? The Mediating Role of the Importance of Parenthood. Br. J. Health Psychol..

[33] Verhaak C.M., Smeenk J.M.J., Evers A.W.M., Kremer J.A., Kraaimaat F.W., Braat D.D.M. (2007). Women’s Emotional Adjustment to IVF: A Systematic Review of 25 Years of Research. Hum. Reprod. Update.

[34] Sathian B., Asim M., Banerjee I., Pizarro A.B., Roy B., van Teijlingen E.R., do Nascimento I.J.B., Alhamad H.K. (2020). Impact of COVID-19 on Clinical Trials and Clinical Research: A Systematic Review. Nepal. J. Epidemiol..

[35] Alviggi C., Esteves S.C., Orvieto R., Conforti A., La Marca A., Fischer R., Andersen C.Y., Bühler K., Sunkara S.K., Polyzos N.P. (2020). COVID-19 and Assisted Reproductive Technology Services: Repercussions for Patients and Proposal for Individualized Clinical Management. Reprod. Biol. Endocrinol..

